# Physical Tests Are Poorly Related to Patient-Reported Outcome Measures during Severe Acute Exacerbations of COPD

**DOI:** 10.3390/jcm11010150

**Published:** 2021-12-28

**Authors:** Kirsten Quadflieg, Ana Machado, Sarah Haesevoets, Marc Daenen, Michiel Thomeer, David Ruttens, Martijn A. Spruit, Chris Burtin

**Affiliations:** 1 REVAL–Rehabilitation Research Center, Faculty of Rehabilitation Sciences, Hasselt University, 3590 Diepenbeek, Belgium; kirsten.quadflieg@uhasselt.be (K.Q.); filipamachado@ua.pt (A.M.); sarah.haesevoets@uhasselt.be (S.H.); 2 BIOMED–Biomedical Research Institute, Hasselt University, 3590 Diepenbeek, Belgium; 3 Respiratory Research and Rehabilitation Laboratory (Lab 3R), School of Health Sciences (ESSUA), University of Aveiro, 3810-193 Aveiro, Portugal; 4 Institute of Biomedicine (iBiMED), University of Aveiro, 3810-193 Aveiro, Portugal; 5 Department Respiratory Medicine, Ziekenhuis Oost-Limburg, 3600 Genk, Belgium; marc.daenen@zol.be (M.D.); michiel.thomeer@zol.be (M.T.); david.ruttens@zol.be (D.R.); 6 Faculty of Medicine and Life Sciences, Hasselt University, 3590 Diepenbeek, Belgium; 7 CIRO, Center of Expertise for Chronic Organ Failure, Department of Research and Education, 6085 NM Horn, The Netherlands; martijnspruit@ciro-horn.nl; 8 Department of Respiratory Medicine, NUTRIM School of Nutrition and Translational Research in Metabolism, Maastricht University Medical Centre, 6229 HX Maastricht, The Netherlands

**Keywords:** chronic obstructive pulmonary disease, acute exacerbations, exercise capacity, muscle function, patient-reported outcome measures

## Abstract

Acute exacerbations of chronic obstructive pulmonary disease (AECOPD) have a negative impact on patients’ health status, including physical function and patient-reported outcomes. We aimed to explore the associations between physical tests and patient-reported outcome measures (PROMs) in hospitalised patients for an AECOPD. Patients were assessed on the day of discharge. Quadriceps force, handgrip strength, short physical performance battery (SPPB), five-repetition sit-to-stand test (5STS), four-meter gait speed test (4MGS), balance test, six-minute walk test (6MWT), COPD Assessment Test (CAT), London Chest Activity of Daily Living scale (LCADL), modified Medical Research Council (mMRC) dyspnea scale, Checklist of Individual Strength (CIS)-fatigue subscale, and Patient Health Questionnaire (PHQ-9) were collected. Sixty-nine patients with an AECOPD were included (54% female; age 69 ± 9 years; FEV_1_ 39.2 (28.6–49.1%) predicted). Six-minute walk distance was strongly correlated with mMRC (*ρ*: −0.64, *p* < 0.0001) and moderately correlated with LCADL total score, subscales self-care and household activities (*ρ* ranging from −0.40 to −0.58, *p* < 0.01). Moreover, 4MGS was moderately correlated with mMRC (*ρ*: −0.49, *p* < 0.0001). Other correlations were weak or non-significant. During a severe AECOPD, physical tests are generally poorly related to PROMs. Therefore, a comprehensive assessment combining both physical tests and PROMs needs to be conducted in these patients to understand their health status.

## 1. Introduction

Chronic obstructive pulmonary disease (COPD) is characterized by a range of symptoms, such as dyspnea, fatigue, depression, and anxiety [1]. In addition, these symptoms are related to diminished exercise capacity and quality of life (QOL) [2,3,4,5].

Acute exacerbations, defined as an acute worsening of respiratory symptoms that requires additional treatment [1], are considered a hallmark characteristic of COPD. Patients require hospitalization in case of a severe exacerbation [1,6]. During hospitalization for an acute exacerbation (AECOPD), functional exercise capacity and quadriceps muscle strength are negatively impacted [7,8,9]. Assessment of physical functioning at the end of an AECOPD-related hospitalization may be difficult in patients with COPD, as they may still suffer from severe dyspnea and fatigue [10,11]. However, this may provide an important indication for early referral for pulmonary rehabilitation [12].

Simple physical tests assessing exercise capacity, functional performance and/or muscle strength are easy to perform and enable us to quantify the impact of a hospitalization on physical function [13]. However, it is not clear to what extent they reflect the impact of the disease from a patient perspective. This is typically measured with patient-reported outcome measures (PROMs) to evaluate symptoms, the impact of symptoms on activities of daily living (ADL), and QOL [14].

Previous studies in patients during the stable phase of COPD found mainly weak to moderate correlations between physical tests and PROMs [15,16] and experts suggest that future studies are needed to examine these associations [17], namely during the acute phase of the disease, where it is known that patients present different characteristics from the stable periods [18].

Therefore, the study aimed to explore the association between physical tests and PROMs in patients admitted to the hospital for an AECOPD. We hypothesized the presence of a correlation between the physical tests and PROMs.

## 2. Materials and Methods

This observational study was approved by The Medical Ethical Committee of Hasselt University and Ziekenhuis Oost-Limburg Genk (Eudract-B-nr: B371201732540, July 2017) and was registered on ClinicalTrials.gov (NCT03250000). Between July 2017 and March 2020, a convenience sample of consecutive patients admitted to the hospital for a doctor-confirmed acute exacerbation, according to the 2018 Global Initiative for Chronic Obstructive Lung Disease (GOLD) guidelines [19], was invited to participate in the study. Exclusion criteria were the presence of other diseases (e.g., unstable cardiac disease, pulmonary hypertension, interstitial lung disease, and malignancies), orthopaedic conditions that impaired functional status, and cognitive and neurological disorders that impaired the ability to comply with study procedures. In addition, all patients gave written informed consent prior to inclusion in the study.

### 2.1. Measurements

Length of hospital stay and number of comorbidities, using the Charlson Comorbidity Index [20], were retrieved from the patients’ file. In addition, post bronchodilator spirometry and lung volumes were performed according to the ATS/ERS guidelines for pulmonary function testing [21,22,23]. This test is embedded yearly in clinical routine, and the most recent stable measurement (i.e., at least three months apart from an AECOPD) was retrieved from patients’ files. Lung function was not measured during the AECOPD-related hospitalization.

Physical tests and PROMs were assessed on the day of discharge. Right isometric quadriceps force was assessed using a hand-held dynamometer (Microfet, Biometrics, NL). Patients were tested while sitting in the upright position. The assessor applied resistance and asked the patient to contract the muscle against the resistance [24]. The test was repeated until reproducible measurements (less than 10% variability) were obtained, and the highest value was used for analyses. The predicted value was also calculated [25]. Right handgrip strength was measured isometrically using a hand-held dynamometer (Jamar, Preston, MI, USA) while sitting in the upright position with the elbow flexed 90 degrees and the wrist in a neutral position [26]. Two maximal efforts were performed. The best was used for analysis, and the predictive value was calculated [27]. The short physical performance battery (SPPB) was assessed, which consists of a four-meter gait speed test (4MGS), a five-repetition sit-to-stand test (5STS), and a balance test [28,29]. For the 4MGS, patients were asked to walk a four-meter track at their usual walking speed, two times. Instructions and test settings were in line with the previously described protocol [30]. During the 5STS, patients were asked to rise from and sit down on a standardized chair with their hands folded across the chest. The test was performed according to the protocol described by Jones et al. [31]. Patients unable to perform the test received a score of 0 or 60 s. A total score of these 3 tests was calculated as the SPPB score, where a score from 0 to 4 could be given to each test and the sum was calculated. A higher total SPPB score (ranging from 0–12 points) means a better physical performance. Functional exercise tolerance was measured using the six-minute walk test (6MWT) performed according to the protocol proposed by ERS/ATS [32]. The test was performed once. The absolute distance walked in the test (6MWD) was used for the analysis, and the predicted value was also calculated [33]. Patients not able to perform the test received a score of 10 m as they could perform the 4MGS test twice.

To assess health-related QOL, the COPD Assessment Test (CAT) was used (0–40 points) [34]. The performance of ADL was evaluated with the London Chest Activity of Daily Living scale (LCADL) (0–75 points), which consists of 4 domains: self-care (0–20 points), domestic activities (0–30 points), physical activities (0–8 points), and leisure time (0–12 points) [35]. To assess symptoms of breathlessness and fatigue, the modified Medical Research Council (mMRC) dyspnoea scale (grade 0–4) and the Checklist Individual Strength (CIS)-fatigue subscale were used (0–56 points), respectively [36,37]. Lastly, the Patient Health Questionnaire (PHQ-9) (0–27 points) was used to screen for depression [38]. Higher scores in all questionnaires represent a lower HRQOL or worse symptoms.

### 2.2. Statistical Analyses

Analyses were performed using JMP PRO 14.2.0. Data are expressed as mean and standard deviation (SD) or median and interquartile range [IQR], depending on the normality of data or percentages. Correlations were analyzed using Pearson’s or Spearman’s correlation, where appropriate. The strength of the association with a correlation coefficients of 0–0.19 was labeled as very weak, 0.20–0.39 as weak, 0.40–0.59 as moderate, 0.60–0.79 as strong, and 0.80–1 as very strong [39]. Significant correlations were corrected for multiple testing using the false discovery rate (FDR) method [40]. Multivariate analyses were performed via multiple linear regression analyses to correct for fixed covariates: sex, age, body mass index (BMI) and forced expiratory volume in 1 s (FEV_1_ %pred). The fixed covariates were chosen as these are easy to assess in clinical practice and related to physical function and patient-reported outcomes [41,42,43,44,45,46].

## 3. Results

A total of 69 patients with severe AECOPD participated in the study. Patient characteristics, physical test outcomes, and PROMs are presented in Table 1.

Correlations between physical tests and PROMs in patients during AECOPD are presented in Table 2. Quadriceps force, handgrip strength, SPPB, and 5STS were only weakly, and mostly non-significantly, correlated with PROMs. The 6MWD was strongly correlated with mMRC score (*ρ*: −0.64, *p* < 0.0001) and moderately correlated with LCADL total score, LCADL subscale self-care, and LCADL subscale household activities (*ρ* ranging from −0.40 to −0.58, *p* < 0.01). A moderate correlation was found between 4MGS and mMRC score (*ρ*: −0.49, *p* < 0.0001). All moderate and strong correlations remained significant after correcting for age, gender, BMI, and FEV_1_ %pred.

## 4. Discussion

This study aimed to investigate the association between physical function and patient-reported outcomes in patients admitted to the hospital for an AECOPD. After adjusting for multiple testing and relevant covariates, we generally observed weak and non-significant correlations between simple functional tests and muscle strength on the one hand and instruments measuring symptoms, the performance of daily life activities, and quality of life on the other hand. However, six-minute walk distance and 4MGS test showed significant moderate to strong correlations with dyspnoea symptoms and ADL performance at hospital discharge.

For the association between physical tests and the subscales of the LCADL questionnaire, we found moderate negative correlations between the 6MWD and the subscales self-care and domestic activities. It is known that patients with COPD experience high metabolic demands and use a high proportion of their ventilation during the performance of domestic ADLs, resulting in higher symptoms perception during these activities [47]. In a recent qualitative paper, patients during an AECOPD stated that being admitted was vital because it was difficult to handle everyday life at home due to breathlessness and exhaustion [48]. These ADLs were mainly assessed in the subscales self-care and domestic activities, which correlated with the 6MWD and can be explained by the high physiological responses on the body and the increase in breathlessness during the performance of this test [49]. No correlation was found between the subscales physical activities and leisure time. The subscale physical activities only address two very different activities (taking stairs and bending), and most patients during an AECOPD were not able to take stairs. The leisure component of the questionnaire attempts to address broader issues of disability, such as the ability to go out socially [50]. During a hospitalization for an AECOPD, patients are more socially isolated and are not able to go out. Therefore, a lack of correlation was found between these subscales and physical tests.

In this study, walking tests are better correlated with some PROMs than tests associated with muscle strength (i.e., analytic muscle assessments and 5STS). When assessing our patients on the day of discharge, they performed an average of 78% and 90% of the predicting value for quadriceps force and handgrip strength, respectively. These are high performances compared to walking distance, where patients had an average of 50% of the predicted value. This could be explained by the different ventilatory demands of these tests. While the tests to assess muscle strength focus on short and powerful contractions, with low ventilatory demands, the 6MWT is a submaximal test with high ventilatory demands that has shown to elicit similar maximal oxygen consumptions as a maximal exercise tests [49]. This suggests that walking, rather than muscle strength, drives the patients’ experience of their health status in this patient group. Besides, it also highlights the need to improve symptoms, exercise capacity, and health-related QOL, showing that early pulmonary rehabilitation could be a beneficial and safe intervention in this population [12].

Most of the associations between physical tests and PROMs were not significant or only weak. The same was found in previous studies, including patients with stable COPD [15,16]. Furthermore, most instruments used to assess patient-reported outcomes are multi-dimensional, which includes domains related to physical functioning and symptoms, and mental, emotional, and social items. Hence, this suggests that the complexity of symptoms experienced by patients with COPD cannot be assessed solely by physical tests.

Nevertheless, in contrast to subjectively measured patient-reported outcomes, simple physical tests can objectively evaluate the impact of hospitalization on physical function [51,52], and their clinical relevance has been linked to morbidity and mortality risk in patients with stable COPD [53,54]. This emphasizes the need for a comprehensive assessment, including both physical tests and PROMs, as they cannot accurately reflect each other accurately. The choice of which physical test and PROM to use, is outside the scope of this article.

### Strengths and Limitations

To our knowledge, this is the first study correlating physical tests and patient-reported outcomes in patients during a severe acute exacerbation of COPD. We corrected for multiple testing and used multivariate analyses to adjust for covariates as this generally improves the efficiency of the analyses and produces more clear evidence of an effect [55]. In addition, the same assessor performed all measurements, suggesting high reliability of the measurements between patients.

Although it is recommended to perform the 6MWT twice, we only assessed it once, as patients’ tolerability was still very limited at the time of hospital discharge. In addition, a score of 60 s was given to patients unable to perform the 5STS, which can be an underrepresentation of the performance.

## 5. Conclusions

During a severe acute exacerbation of COPD, physical tests are poorly related to patient-reported outcome measures and likely provide complementary information. A comprehensive assessment combining both physical tests and PROMs needs to be conducted in these patients. Future studies should focus on the development of a core outcome set, including both physical tests and PROMs in this patient population.

## Figures and Tables

**Table 1:** Patient characteristics, physical test outcomes, and patient-reported outcome measures.

	Patients during an Acute Exacerbation *n* = 69
**Patient characteristics**	
Female (%)	37 (54)
Age (years)	69 ± 9
BMI (kg/m²)	25.3 ± 5.6
LOS (days)	5 (4–8)
CCI (score)	4 (3–5)
FEV_1_ (L)	0.92 (0.70–1.30)
FEV_1_ (% predicted)	39.2 (28.6–49.1)
FVC (L)	2.10 ± 0.69
FVC (% predicted)	65.1 (51.5–74.2)
FEV_1_/FVC (%)	48.8 ± 11.5
**Physical test outcome measures**	
Quadriceps force (N)	240 ± 97
Quadriceps force (% predicted)	78.5 ± 26.0
Handgrip strength (kg)	27 ± 9
Handgrip strength (% predicted)	90.8 ± 20.2
SPPB total score (points)	9 (7–11)
5STS (s)	14 (10–21)
4MGS (m/s)	0.70 ± 0.24
6MWD (m)	237 ± 116
6MWD (% predicted)	49.6 ± 24.3
BORG dyspnea end (points)	5 (3–7)
BORG fatigue end (points)	3 (0–5)
**Patient-reported outcomes**	
CAT score (points)	22 ± 7
LCADL total score (points)	38 ± 14
Self-care (points)	10 (8–15)
Domestic activities (points)	17 (9–24)
Physical activities (point)	5 (5–7)
Leisure time (points)	6 (4–7)
mMRC dyspnea (grade)	3 (2–4)
CIS-Fatigue score (points)	46 (38–53)
PHQ-9 score (points)	7 (4–11)

Data are presented as mean ± SD, or median (interquartile range), depending on normality of data, or as *n* (%). Lung function measurement was performed during a stable disease phase prior to the exacerbation. Abbreviations: BMI: body mass index; LOS: length of hospital stay; CCI: Charlson Comorbidity Index; FEV_1_: forced expiratory volume in 1 s; FVC: forced vital capacity; SPPB: short physical performance battery; 5STS: five-repetition sit-to-stand; 4MGS: four-meter gait speed test; 6MWD: six-minute walk distance; CAT: COPD Assessment Test; LCADL: London Chest Activity of Daily Living scale; mMRC: modified Medical Research Council; CIS: Checklist Individual Strength; PHQ-9: Patient Health Questionnaire.

**Table 2:** Correlation between physical test outcomes and patient-reported outcome measures.

	Quadriceps Force (N)	Handgrip Strength (kg)	6MWD (m)	SPPB (Points)	5STS (s)	4MGS (m/s)
CAT score (points)	−0.14 _r_	−0.22 _r_	−0.10 _r_	0.01 *_ρ_*	−0.07 *_ρ_*	−0.16 _r_
LCADL total score (points)	−0.25 _r_	−0.19 _r_	−0.45 _r_^a,b^	−0.27 *_ρ_*	0.18 *_ρ_*	−0.37 _r_^a,b^
Self-care score (points)	−0.12 *_ρ_*	−0.05 *_ρ_*	−0.43 *_ρ_*^a,b^	−0.30 *_ρ_*^a,b^	0.15 *_ρ_*	−0.37 *_ρ_*^a,b^
Domestic activities score (points)	−0.34 *_ρ_*^a^	−0.30 *_ρ_*^a^	−0.42 *_ρ_*^a,b^	−0.27 *_ρ_*^a^	0.23 *_ρ_*	−0.31 *_ρ_*
Physical activities score (points)	0.13 *_ρ_*	0.20 *_ρ_*	0.08 *_ρ_*	0.08 *_ρ_*	−0.10 *_ρ_*	0.03 *_ρ_*
Leisure time score (points)	−0.05 *_ρ_*	−0.01 *_ρ_*	−0.16 *_ρ_*	0.03 *_ρ_*	−0.08 *_ρ_*	−0.10 *_ρ_*
mMRC dyspnea (grade)	−0.22 *_ρ_*	−0.23 *_ρ_*	−0.64 *_ρ_*^a,b^	−0.21 *_ρ_*	0.13 *_ρ_*	−0.49 *_ρ_*^a,b^
CIS-Fatigue score (points)	−0.25 *_ρ_*	−0.21 *_ρ_*	−0.25 *_ρ_*	−0.02 *_ρ_*	−0.10 *_ρ_*	−0.22 *_ρ_*
PHQ-9 score (points)	−0.07 *_ρ_*	−0.11 *_ρ_*	−0.08 *_ρ_*	0.07 *_ρ_*	−0.09 *_ρ_*	0.01 *_ρ_*

Correlations are presented using Pearson’s (r) or Spearman’s (*ρ*) correlation; a = significant correlation after correcting for multiple testing; b = significant correlation after correcting for covariates. Abbreviations: 6MWD: six-minute walk distance; SPPB: short physical performance battery; 5STS: five-repetition sit-to-stand test; 4MGS: four-meter gait speed test; CAT: COPD Assessment Test; LCADL: London Chest Activity of Daily Living scale; mMRC: modified Medical Research Council; CIS: Checklist Individual Strength; PHQ-9: Patient Health Questionnaire.

## Data Availability

Not applicable.
